# [Expression of Concern] Involvement of Cyr61 in the growth, invasiveness and adhesion of esophageal squamous cell carcinoma cells

**DOI:** 10.3892/ijmm.2026.5811

**Published:** 2026-03-24

**Authors:** Jian-Jun Xie, Li-Yan Xu, Yang-Min Xie, Ze-Peng Du, Cai-Hua Feng, Hui Dong, En-Min Li

Int J Mol Med 27: 429-434, 2011; DOI: 10.3892/ijmm.2011.603

Following the publication of the above paper, it was drawn to the Editor's attention by a concerned reader that, regarding the cell migration assay data shown in Fig. 4A on p. 432, the 'EC109' and 'Vector' data panels were found to contain an overlapping section, such that data which were intended to show the results of differently performed experiments were apparently derived from the same original source.

The authors have been contacted by the Editorial Office to offer an explanation for the apparent anomaly in the presentation of the data in their paper, and we are awaiting their response. Owing to the fact that the Editorial Office has been made aware of potential issues surrounding the scientific integrity of this paper, we are issuing an Expression of Concern to notify readers of this potential problem while the Editorial Office continues to investigate this matter further.

